# VCP represses pathological cardiac hypertrophy

**DOI:** 10.18632/aging.101357

**Published:** 2017-12-26

**Authors:** Ning Zhou, Shaunrick Stoll, Hongyu Qiu

**Affiliations:** Division of Physiology, Department of Basic Sciences, School of Medicine, Loma Linda University, Loma Linda, CA 92354, USA

**Keywords:** valosin-containing protein, cardiac hypertrophy, pressure overload

Pressure overload-induced cardiac hypertrophy, such as that caused by chronic hypertension, is a major independent risk factor for heart failure and is a leading cause of the increasing incidence of cardiac morbidity and mortality in the elder [1]. Accumulating evidences from studies in patients and animal models suggest that cardiac hypertrophy induced by chronic pressure overload is not a compensatory but rather a maladaptive process [2]. Despite intensive research efforts over several decades, the molecular mechanisms of hypertrophic heart failure are not fully understood. Therefore, it has become compulsory to identify novel targets involved in the pathogenesis of cardiac hypertrophy and its transition to heart failure.

The valosin-containing protein (VCP), also known as Cdc48 in yeast and plants, CDC-48 in worms and Ter94 in flies, is a member of the type II AAA (ATPases Associated with various cellular Activities) family which is ubiquitously expressed in cells [3]. VCP correlates with cell growth and survival in cancer cells and has been implicated in multisystem degenerative disorders [4]. Our previous studies identified VCP in the heart and showed that it is a critical mediator of cardiomyocyte survival under ischemic stress both *in vitro* and *in vivo* [5, 6]. However, the role of VCP in cardiac growth or hypertrophy under stress conditions was completely unknown.

In our recent study [7], we observed that VCP expression was significantly down-regulated in the hypertrophic left ventricle (LV) tissues of both hypertensive rats and transverse aortic constriction (TAC)-induced pressure-overloaded mice. These findings demonstrated a strong link between down-regulation of VCP expression and hypertensive cardiomyopathy. Reciprocally, cardiac-specific over-expression of VCP in a transgenic (TG) mouse signi-ficantly attenuated the pressure overload-induced cardiac hypertrophy. These data together suggested that VCP plays a critical role in pressure overload-induced cardiac hypertrophy. Direct evidence of VCP's cardio-protective effect was shown in an *in vitro* study where VCP was downregulated in AngII-induced hypertrophic cardiomyocytes in a dose- and time-dependent manner, whereas the overexpression of VCP prevented AngII-induced cardiomyocyte hypertrophy.

These data indicated that VCP resists the neurohumoral-stimulated hypertrophic response in cardiomyocytes. In addition, we also found that VCP plays a dual role on the regulation of the mechanistic target of rapamycin (mTOR) signaling in the heart: activating the survival-promoting mTOR complex 2 (mTORC2) but repressing the stress-induced growth-promoting mTOR complex 1 (mTORC1). Under pressure overload, a reduction of VCP was accompanied by an increase of phosphorylation of AKT at Thr308 (pAKT T308) and an elevated mTORC1/S6K activity in hypertrophic LVs in TAC WT mice. On the contrary, overexpression of VCP significantly suppressed this signaling in TAC VCP TG mice. These data suggested that VCP acts as a negative regulator of mTORC1 under stress of pressure overload. Our results also demonstrated that VCP is able to activate AKT phosphorylation at Serine 473 (pAKT S473). Although the underlying mechanisms are not yet clear, given the evidence from our study that VCP is able to activate AKT S473 independent of the activation of PI3K, one possibility is that VCP activates AKT S473 via the mTORC2, which is another known activator of pAKTS473. Furthermore, since VCP activates AKT S473 but not AKT T308 at baseline, indicating that VCP mediates the mTORC2 effect prior to the mTORC1 effect, it is reasonable to presume that the inhibition of pAKT T308 after TAC in VCP TG mice may result from a secondary effect of the activation of mTORC2 by VCP. These selective effects of VCP on mTORC1 and mTORC2 are different from that of other mTOR regulators identified in the heart, such as rapamycine, and also distinct from the function of VCP observed in other tissues. Moreover, VCP suppressed mTORC1 signaling only under the stress of TAC but not at the baseline condition. This characteristic of VCP further highlights its clinical significance due to its selective effects on pathological conditions without affecting physiological cardiac growth and function.

**Figure 1:**
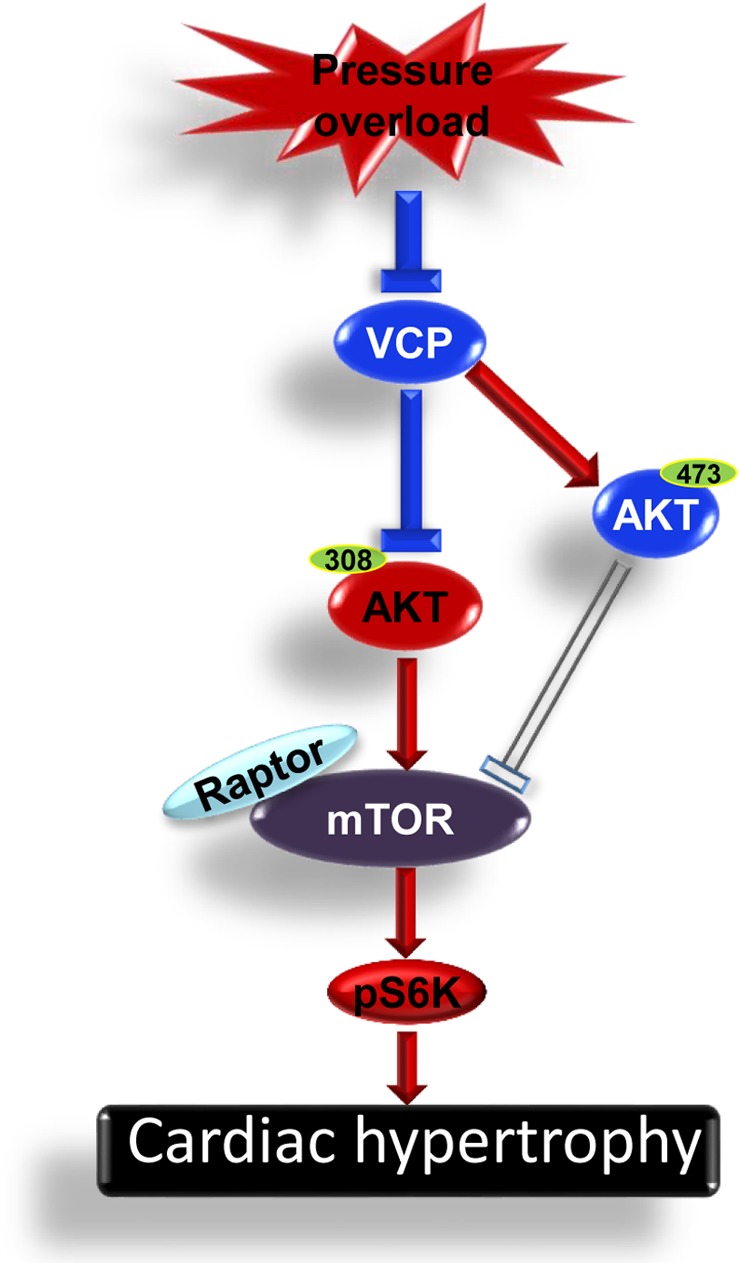
An illustration of the mechanism by which VCP protects against pressure overload induced cardiac hypertrophy Pressure overload reduces VCP expression which attenuates the inhibitive effect of VCP on AKT/mTORC1/S6K signaling, subsequently promotes the cardiomyocyte hypertrophy.

As shown in the Figure 1, our data collectively concluded that pressure overload reduced VCP expression in the heart which attenuated the inhibitive effect of VCP on mTORC1/S6K signaling, subsequently promoting the pro-growth pathway and resulting in cardiac hypertrophy. These findings bring new insights to the regulatory effects of VCP in the heart and also lead to a new therapeutic target for pressure overload-induced cardiac pathogenesis, which is directly relevant to the condition of chronic hypertension in old individuals. Our results will also stimulate further investigation for a deep mechanistic understanding of the selective effects of VCP on mTOR signaling and the role of this pathway in both physiological and pathological conditions.
